# Molecular Mechanisms of Cereblon-Interacting Small Molecules in Multiple Myeloma Therapy

**DOI:** 10.3390/jpm11111185

**Published:** 2021-11-11

**Authors:** Matteo Costacurta, Jackson He, Philip E. Thompson, Jake Shortt

**Affiliations:** 1 Blood Cancer Therapeutics Laboratory, Department of Medicine, School of Clinical Sciences at Monash Health, Monash University, Clayton, VIC 3168, Australia; 2 Monash Haematology, Monash Health, Clayton, VIC 3168, Australia; 3 Medicinal Chemistry, Monash Institute of Pharmaceutical Sciences, Monash University, 381 Royal Parade, Parkville, VIC 3052, Australia

**Keywords:** thalidomide, IMiDs, CELMoDs, Cereblon, multiple myeloma

## Abstract

Thalidomide analogues (or immunomodulatory imide drugs, IMiDs) are cornerstones in the treatment of multiple myeloma (MM). These drugs bind Cereblon (CRBN), a receptor for the Cullin-ring 4 ubiquitin-ligase (CRL4) complex, to modify its substrate specificity. IMiDs mediate CRBN-dependent engagement and proteasomal degradation of ‘neosubstrates’, Ikaros (IKZF1) and Aiolos (IKZF3), conveying concurrent antimyeloma activity and T-cell costimulation. There is now a greater understanding of physiological CRBN functions, including endogenous substrates and chaperone activity. CRISPR Cas9-based genome-wide screening has further elucidated the complex cellular machinery implicated in IMiD sensitivity, including IKZF1/3-independent mechanisms. New-generation IMiD derivatives with more potent anti-cancer properties—the CELMoDs (Cereblon E3 ligase modulators)—are now being evaluated. Rational drug design also allows ‘hijacking’ of CRL4^CRBN^ utilising proteolysis targeting chimeras (PROTACs) to convey entirely distinct substrate repertoires. As all these chemotypes—thalidomide, IMiDs, CELMoDs and PROTACs—engage CRBN and modify its functions, we describe them here in aggregate as ‘CRBN-interacting small molecules’ (CISMs). In this review, we provide a contemporary summary of the biological consequences of CRBN modulation by CISMs. Detailed molecular insight into CRBN–CISM interactions now provides an opportunity to more effectively target previously elusive cancer dependencies, representing a new and powerful tool for the implementation of precision medicine.

## 1. Introduction

Thalidomide analogues, such as lenalidomide and pomalidomide, have significantly improved outcomes for patients with multiple myeloma (MM), an incurable malignancy of the plasma cell [1,2]. Prior to the precise elucidation of their molecular mechanism of action, thalidomide analogues were characterized by anti-inflammatory and immune-enhancing effects and designated as immunomodulatory imide drugs (IMiDs). It is now apparent that many of the biological activities of IMiDs are mediated by their protein target, Cereblon (CRBN). The *CRBN* gene is located on chromosome 3p and encodes a 50kDa LON protease which regulates a plethora of biological roles. Before CRBN was identified as the protein target of IMiDs, a *CRBN* germline nonsense mutation was implicated in an autosomal recessive form of mental retardation [3,4]. CRBN functions as a receptor for the Cullin Ring Ligand 4 E3 ubiquitin ligase complex (CRL4^CRBN^), where Cullin 4 (CUL4A/B) is the scaffold, Ring-Box 1 (RBX1) is the RING component binding the E2 enzyme, and DNA Damage Binding protein 1 (DDB1) is the adaptor [5,6]. CRBN docks onto DDB1 with its helical bundle domain and engages substrates for ubiquitination via its C-terminus domain (CTD). When bound by an IMiD, CRBN’s E3 ligase specificity is redirected towards non-physiological proteins targets which are subsequently ubiquitinated and/or degraded [7,8]. It is also evident that IMiDs modulate non-E3 ligase-related properties of CRBN, such as chaperone functions [9,10]. More recently, the term ‘CELMoDs’ (Cereblon E3 ligase modulators) was coined to refer to a ‘new generation’ of IMiDs that have been developed in the post-CRBN era to exploit improved biological activities [11,12,13]. Moreover, bespoke chimeric compounds combining a targeted small molecule with a phthalimide moiety can ‘hijack’ CRL4^CRBN^ to degrade entirely distinct substrates [14,15,16,17]. Here, we review the physiological activities of CRBN and how these may be modulated by CRBN-interacting small molecules (CISMs) to improve precision in the treatment of hematological malignancies.

## 2. Mechanisms of Immunomodulatory Imide Drug (IMiD) Activity in Multiple Myeloma

The biological changes induced by IMiD treatment can be divided into tumor cell-extrinsic effects (e.g., relating to the host immune system and bone marrow microenvironment) and tumor cell-intrinsic effects (i.e., direct toxicity to the malignant plasma cell). The earliest cell-based studies of thalidomide and its analogues exploited modulation of immune effector activities including the suppression of tumor necrosis factor (TNF)-α secretion by mononuclear cells following lipopolysaccharide (LPS) challenge [18,19,20] and the capacity to costimulate T-cells [21]. In addition to stimulating cytotoxic effector cells, IMiDs increase antigen presentation by dendritic cells and suppress the activity of T regulatory lymphocytes [22,23,24,25,26,27,28,29]. IMiDs have diverse effects on the bone marrow microenvironment including anti-angiogenic activity, bone protective properties and altered cellular adhesion. Thalidomide impairs angiogenesis via suppression of vascular endothelial growth factor (VEGF) signaling [30,31]. Lenalidomide also impairs angiogenesis by a reduction in VEGF expression, together with inhibition of fibroblast growth factor (FGF) and interleukin (IL)-6 signaling, and shows increased anti-angiogenic activity relative to thalidomide [32,33,34]. Amelioration of myeloma-related bone disease is a consequence of reduced Receptor Activator of NFκB Ligand (RANKL) expression and osteoclast activity [35]. Cell adhesion to the bone marrow extracellular matrix and interactions with stromal cells are mediated by adhesion molecules such as integrins and their ligands Intercellular Adhesion Molecule (ICAM)-1 and Vascular Cell Adhesion Molecular (VCAM)-1 [36]; IMiDs suppress the expression of these molecules and, therefore, interfere with pro-survival paracrine signaling [36]. The net result is disruption of the protective effects of the bone marrow niche with concurrent augmentation of the anti-myeloma immune response.

Cell-intrinsic effects on the myeloma cell include cytostasis and induction of apoptosis. IMiDs cause G_0_–G_1_ cell cycle arrest by the induction of tumor suppressors p21, p27, and members of the Early Growth Response (EGR) protein family [37], together with the inhibition of cyclin-dependent kinases-2, -4 and -6 [38,39]. Thalidomide also suppresses Nuclear Factor kappa of activated B-cells (NF-κB) signaling [40] which has apoptotic effects consequent to downregulation of B-cell lymphoma 2 (BCL2) and BCL2-like proteins with the release of mitochondrial cytochrome c [41]. Contemporary understanding of IMiD biology indicates that these tumor cell intrinsic effects, and the broader microenvironmental and immunomodulatory activities, are underpinned by a direct interaction with CRBN.

## 3. Cereblon (CRBN) Is Required for the Anti-Myeloma Activity of IMiDs

A major breakthrough in deciphering the molecular mechanism of IMiDs came with the discovery that thalidomide bound directly to CRBN and that this interaction was necessary for its teratogenic effects using zebrafish and chicken embryo modelling [5]. Soon after, it was shown that CRBN expression was required for the antimyeloma activity of IMiDs [42]. The primacy of CRBN in the IMiD mechanism of action has now been established by examining the cellular changes that arise in the context of IMiD resistance. Prolonged in vitro exposure to increasing concentrations of lenalidomide resulted in IMiD-resistant myeloma cells with reduced CRBN expression caused by deletion of one copy of *CRBN* [42]. CRBN levels are positively associated with clinical responses to lenalidomide [43] and *CRBN* expression is lower in plasma cells from patients relapsing after lenalidomide therapy [42]. Splice-out of *CRBN* exon 10, corresponding to the thalidomide binding domain (TBD), has been associated with lenalidomide refractoriness [44]. Recent deep sequencing work has demonstrated that point mutations, copy number loss and structural variants of *CRBN* can impair responses to lenalidomide and pomalidomide, and that these aberrations significantly reduce the survival of MM patients [45]. Together these data indicate that *CRBN* downregulation and/or mutations appear to represent the main mechanism by which MM cells escape the anti-cancer effects of IMiDs in vivo. 

## 4. CRL4^CRBN^ Neosubstrates in Disease Responses and Teratogenicity

**Figure 1:**
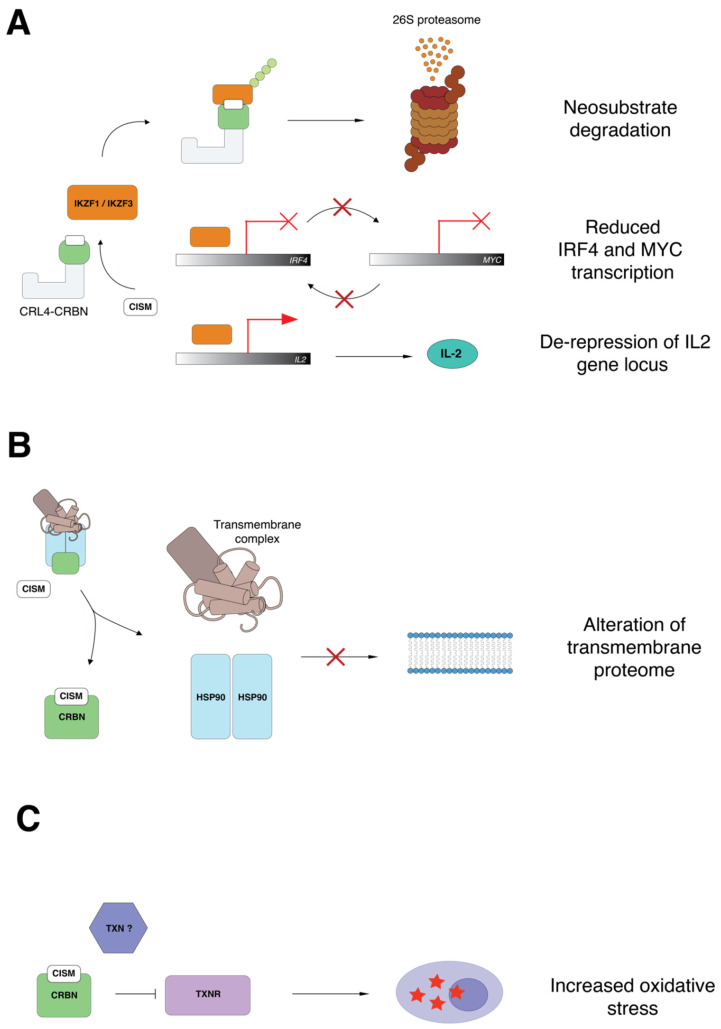
Pathways that are modulated by immunomodulatory imide drugs (IMiDs) with therapeutic significance in multiple myeloma (MM). (**A**) Proteasomal degradation of Ikaros (IKZF1) and Aiolos (IKZF3) following IMiD treatment results in suppression of the *IRF4-MYC* transcriptional feedback loop. (**B**) CRBN is an HSP90 co-chaperone which facilitates the folding of transmembrane proteins, potentially modulating cell-to-cell interactions in the bone marrow microenvironment. (**C**) Lenalidomide binds Cereblon (CRBN) and interferes with neutralization of reactive oxygen species by disrupting the thioredoxin (TXN)-thioredoxin reductase (TXNR) axis.

In 2014, two seminal papers demonstrated that lenalidomide’s interaction with CRBN changes its substrate specificity to induce the proteasomal-dependent degradation of Ikaros (or IKZF1) and Aiolos (or IKZF3) [7,8]. IKZF1 and IKZF3 were thus defined CRBN ‘neosubstrates’ because they only become CRBN targets in the presence of an IMiD. Together IKZF1 and -3 are critical transcription factors within the hematological compartment. Degradation of IKZF1/3 is associated with downregulation of interferon regulatory factor 4, *IRF4* [7], an ‘oncogene of addiction’ in MM [46]. IRF4 sustains the expression of *MYC*, another important myeloma oncogene (Figure 1A) [47]. Shaffer and colleagues demonstrated that MYC enhances the expression of IRF4, such that IRF4 and MYC reciprocally promote each other’s transcription within a positive feedback loop [46]. More recent work defined IKZF1/3, IRF4 and MYC as enhancer-associated transcription factors sustaining the core transcriptional regulatory network of the malignant plasma cell [48]. Disruption of the IKZF1/3-IRF4-MYC transcriptional axis is of specific importance in MM [39,49], as studies have shown that in other diseases, such as primary effusion lymphoma, degradation of IKZF1/3 is uncoupled from lenalidomide-induced suppression of IRF4 and MYC [50]. IKZF1/3 also transcriptionally repress the *IL2* gene and IKZF1/3 degradation evokes IL-2 production (Figure 1A) [26,51]. This is thought to be one of the mechanisms by which IMiDs mediate T-cell costimulation.

In addition to MM and certain other mature B-cell neoplasms, lenalidomide is indicated for the treatment of myelodysplasia (MDS) with deletion of chromosome 5q (del(5q) or 5q minus syndrome). Patients with del(5q) typically present with refractory anemia and thrombocytosis [52]. The pathogenic mechanism implicates haploinsufficiency of ribosomal protein (RP) S14, which resides within the del(5q) common deleted region (CDR), as the cause of a p53-dependent anemic phenotype [53,54,55]. Haploinsufficiency of another CDR encoded gene, microRNA-145, is associated with increased expression of the transcription factor Friend Leukemia Integration (FLI)-1 which skews hematopoiesis towards megakaryopoiesis, resulting in thrombocytosis [56]. Lenalidomide treatment mitigates both anemia and thrombocytosis in 5q minus syndrome, but it is unclear how lenalidomide directly impacts the activity of RPS14 and microRNA-145 [57]. The specific activity of lenalidomide in MDS with del(5q) is now explained by its capacity to target a further CDR encoded protein, casein kinase 1 alpha (CK1α), for proteasomal degradation [58]. Reduced expression of CK1α in the del(5q) clone conveys sensitivity to the apoptotic effects of lenalidomide via stabilization of p53. Deletion or pharmacological inhibition of CK1α is also detrimental to MM cells, indicating that lenalidomide-dependent degradation of CK1α may contribute to its anti-myeloma activity [59,60,61]. 

**Figure 2:**
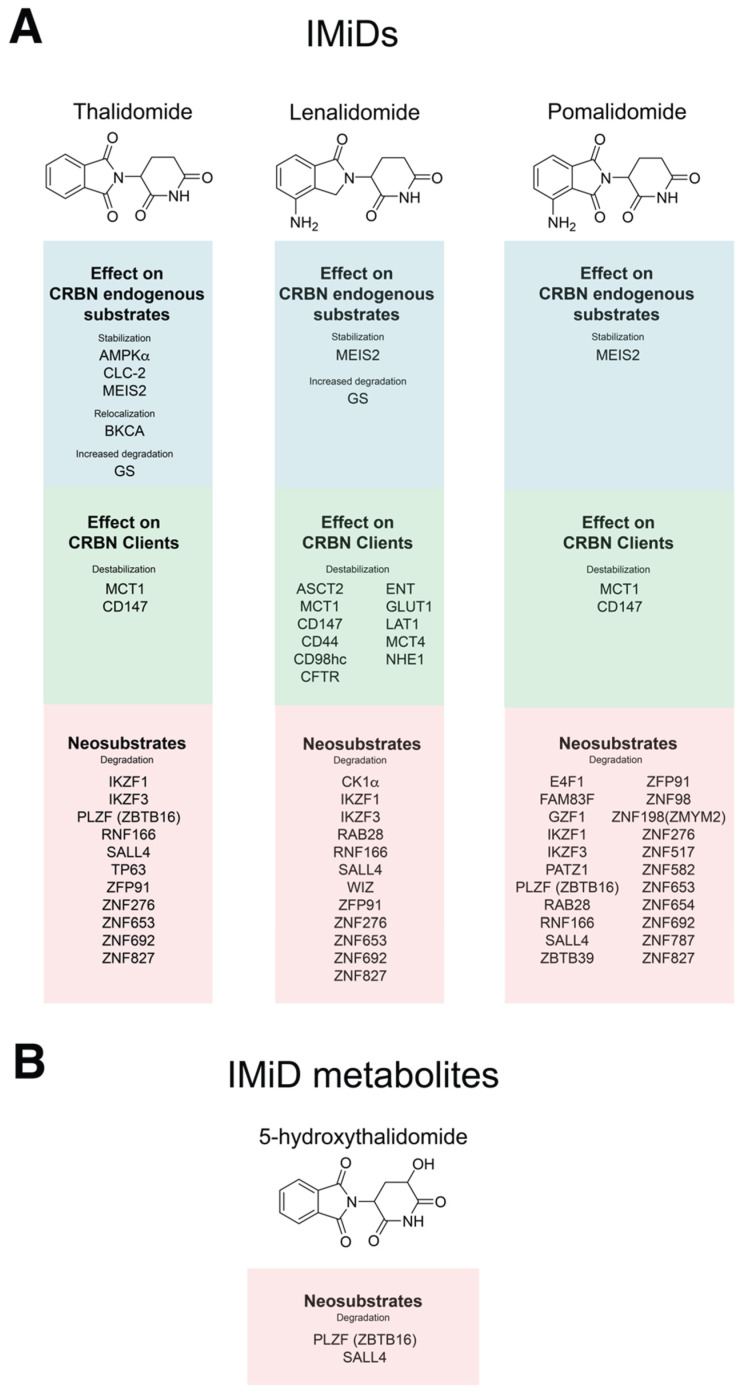
Structure of IMiDs and their reported substrate repertoires. (**A**) Chemical structures of thalidomide, lenalidomide and pomalidomide. Endogenous CRBN substrates that are stabilized or re-localized upon treatment with each of these molecules are listed in the blue panels. CRBN clients, whose maturation/stability is modulated by the respective IMiDs are listed in the green panels. CRBN neosubstrates are listed within the red panels. (**B**) The structure of thalidomide metabolite, 5-hydroxythalidomide and its demonstrated neosubstrates.

Greater understanding of the IMiD mechanism of action has facilitated the discovery of an expanding repertoire of CRBN neosubstrates (Figure 2A). More recently disclosed neosubstrates include ZFP91, SALL4 and PLZF. These proteins may be differentially regulated by the emerging suite of CRBN-interacting small molecules (IMiDs and CELMoDs; discussed below). ZFP91 is a zinc-finger (ZF) putative ubiquitin-ligase and CRBN neosubstrate [62] that regulates NF-κB signaling by activating NFkB-inducing kinase (NIK) via Lys63 ubiquitination [43]. ZFP91 is also associated with maintenance of T-regulatory cell homeostasis [44]. However, the contribution of ZFP91 degradation to the anti-myeloma activity of IMiDs remains undefined. Mutation of *SALL4* causes Duane radial ray syndrome, a congenital disorder resulting in abnormalities of the bones of the forelimbs. SALL4 is degraded upon thalidomide treatment, indicating a potential mechanism of thalidomide teratogenicity [63]. In related work, other neosubstrates belonging to the C_2_H_2_ zinc-finger protein family (e.g., RNF166, FAM83F, GZF1, ZBTB39) were identified as potentially contributing to IMiD-induced embryopathy. PLZF/ZBTB16 is another ZF protein that is degraded following thalidomide treatment, and loss of PLZF has been linked to limb abnormalities [64]. Finally, thalidomide-dependent degradation of p63 has been implicated in the pathogenesis of thalidomide embryopathy [65]. Taken together, these studies suggest that abnormal fetal development may be caused by the modulation of multiple CRBN neosubstrates. 

## 5. Structure–Activity Relationship of CRL4^CRBN^ in Complex with CRBN-Interacting Small Molecules (CISMs)

Thalidomide’s chemical structure is represented by a glutarimide ring bonded to a phthalimide group [6,66]. The glutarimide ring binds CRBN at the TBD, which contains a tryptophan-rich pocket (Tryptophan 380, 386, 402 and Phenylalanine 404) and is located at the CRBN CTD [6]. The substitution of Tyr384 and Trp386 with alanine residues abrogates the binding of CISMs into this tri-Tryp pocket [5]. Modifications of functional groups (e.g., NH_2_ on C4 of the phthalimide substituent in pomalidomide or on the isoindolinone moiety in lenalidomide) account for differences in the spatial interaction between different CISMs and CRBN. Such differences likely explain differential neosubstrate specificity between chemotypes [6]. Thalidomide is a racemic compound, and the *S*-enantiomer displays greater CRBN binding relative to the *R*-enantiomer. *S*-thalidomide is thought to be responsible for teratogenic effects [66] and it is unclear whether *R*-thalidomide engages different CRBN neosubstrates to those recruited by *S*-thalidomide [66]. The potential role of thalidomide metabolites in neosubstrate degradation has also been evaluated. Degradation of PLZF and SALL4 is mediated by both thalidomide and 5-hydroxythalidomide (5-HT), but IKZF1 is only degraded by thalidomide itself [64]. Interestingly, 5-HT was more efficient in degrading SALL4 than unmodified thalidomide (Figure 2B). Therefore, substrate selectivity in vivo may relate to the activities of both parent molecules and metabolites deriving from newer CISMs, including IMiDs and CELMoDs, and this activity spectrum remains poorly determined. A comprehensive study of the C_2_H_2_ ZF proteome expanded the pool of known CRBN neosubstrates and highlighted the importance of specific ZF domains in determining the engagement of neosubstrates upon treatment with different IMiDs [67]. In particular, the ZF2 domain was identified as the necessary element required for degradation of C_2_H_2_ ZF proteins, and the presence of ZF3 increased the affinity between CRBN and pomalidomide. Structural studies of CK1α in complex with CRBN and lenalidomide have, however, highlighted that degradation of non-ZF proteins, such as CK1α, can still be engaged by a CISM despite the lack of ZF domains [58,68]. This observation demonstrates that engagement of neosubstrates is not exclusively dependent on the presence of a ZF domain but is determined by the three-dimensional structure of the binding interface between CRBN/CISM and its target. 

## 6. Genome-Scale CRISPR Screening as a Tool for Identifying Mediators of Sensitivity to IMiDs

CRISPR-Cas9 technology facilitates the editing of individual genes within a population of cells transduced with viral library expressing short guide (sg)-RNAs covering the entire genome. Exposure to a selective pressure (e.g., a drug) for a defined period enables positive selection of drug-resistant clones and dropout of sensitized clones [69,70,71,72]. Employment of *in vitro* CRISPR genome-wide screening has identified the minimal machinery required for the response to the IMiDs in addition to CRBN itself [73,74,75]. Together with essential components of the CRL4 ligase, loss of genes related or unrelated to protein degradation can result in IMiD resistance. *CRBN, DDB1, RBX1, GLMN, UBE2G1, UBE2M, UBE2D3, CAND1* and genes encoding subunits of the Constitutive Photomorphogenesis (COP)-9 signalosome (*COPS1, COPS2, COPS3, COPS4, COPS5, COPS6, COPS7A, COPS7B, COPS8*) are involved in ubiquitination and degradation of IMiD neosubstrates. UBE2G1, UBE2D3 are E2 enzymes implicated in the process of ubiquitin-tagging of substrates [76], whilst UBE2M promotes NEDDylation of CUL4 [77]. The COP9 signalosome (CSN) is a 9-subunit complex which removes NEDD8 from Cullin scaffolds [78,79]. NEDD8 is required for ubiquitination of CRL4 substrates and CSN-dependent removal or inhibition with the small molecule MLN4924 inhibits this process [77]. Once a target has been ubiquitinated by CRL4^CRBN^, removal of NEDD8 and disassembly of CRL4^CRBN^ by CAND1 are required for recycling and reassembly of the complex. Loss of any of these factors interferes with the cycling process of assembly-ubiquitination-disassembly of the CRL4^CRBN^ ligase, disrupting protein ubiquitination and causing resistance to IMiD treatment [73]. The CSN is also regulates SCF^Fbxo7^, an E3 ligase which targets CRBN itself; its activity inhibits the degradation of CRBN by SCF^Fbxo7^ and CSN loss causes a reduction in CRBN [74]. This led to the hypothesis that the synergy between lenalidomide and proteasome inhibitors in the clinic is based on sequential scheduling, where the proteasome inhibitor is given before lenalidomide and raises baseline CRBN levels by inhibiting SCF^Fbxo7^ mediated CRBN degradation [74]. 

Other genes that are not directly implicated in protein turnover are also required for IMiD sensitivity. Loss of any of these genes—*NCOR1, EDC4, RARA, SNRNP25, OTUB1, PLAA, DEPDC5, SRP14, XRN1, EIF4A1, ARID2, SCAP, MBTPS1, MBPTS2, C12orf49, HIST1H4F*-caused IMiD resistance [73,74,75]. Our laboratory performed genome-wide CRISPR screening in cells with acquired lenalidomide resistance [42], aiming to identify potential candidates for IMiD resensitization. Here we found that deletion of *TOP2B, ATXN7, MIER3* and subunits of the glucose-responsive GID E3 ligase complex (*YPEL5, MAEA*) restored IMiD sensitivity [75]. TOP2B is of potential therapeutic relevance as it can be pharmacologically targeted by the cardioprotective agent, dexrazoxane and the topoisomerase poison, etoposide. Interestingly, CRBN plays a role following DNA damage evoked by etoposide in non-tumor cells as CRBN disrupts signaling between TP53 and members of the BCL2 family, resulting in resistance to apoptosis following a DNA insult [80]. Others have demonstrated IMiD resensitization by inhibition of STAT3 signaling or the EP300/CBP acetyltransferase [81]. Inhibition of EP300/CBP causes acute repression of *IRF4* and *MYC* in myeloma cells [82], further indicating that MM cells are strongly dependent on these two key transcription factors.

## 7. CRBN Functions as a Molecular Chaperone

In 2016, it was discovered that CRBN interacts with MCT1 (or SLC16A1) and CD147 (Basigin) [10]. These proteins have several physiological roles such as nutrient transport, migration of inflammatory cells and induction of metalloproteinases [56]. CRBN promotes maturation of MCT1 in complex with CD147 independent of ubiquitination, and the binding of CRBN to CUL4 or MCT1-CD147 is mutually exclusive. IMiDs inhibit the maturation of MCT1-CD147 in a dose-dependent manner and suppress pro-survival signaling. This important additional mechanistic data provides evidence for an alternative pathway by which IMiDs exert anti-tumor and microenvironmental effects. More recently, the same group reported that CRBN cooperates with the chaperone proteins HSP90 and AHA1 to stabilize the transmembrane protein CD98h-LAT1; this work demonstrated that all CRBN client misfolded proteins are also HSP90 clients, and these are directed to the surface membrane (Figure 1B) [9]. Such clients included CD44, MCT1, MCT4, ENT, CD147, LAT1, CD98h, GLUT1, NHE1, ASCT2 and CFTR (Figure 2A). In a similar manner to that of MCT1-CD147, CD98h-LAT1 was found to be a vulnerability in MM with the advantage of being exploitable also as a radiotheranostic target [9]. Although IMiD-mediated destabilization of MCT1-CD147 has been defined as potentially teratogenic, it remains unclear whether destabilization of CD98h-LAT1 has an impact on fetal development.

## 8. Other Physiological and Pathological Roles of CRBN

*CRBN* was initially identified as a gene implicated by mutation in a mental retardation syndrome [4]. The *CRBN^R419X^* mutation causes expression of a truncated transcript that escapes non-sense mediated decay. This alteration was associated with disturbances of Ca^++^ and K^+^ conductance resulting in increased reactivity to intracellular Ca^++^ [3]. Other CRBN substrates are now implicated in the regulation of membrane electrical potential (Figure 2A). BKCA channels, which regulate Ca^2+^-induced release of neurotransmitters and neuronal excitability, are ubiquitinated by CRL4^CRBN^ [83]. Interestingly, these are not then degraded by the proteasome but are instead retained in the endoplasmic reticulum. Upon loss of CRBN or thalidomide exposure, these BK channels are subsequently redirected to the cell surface conveying increased neuronal excitability. The voltage gated CLC chloride channels CLC-1 and -2 have also been identified as CRBN targets. These are engaged by CRBN for polyubiquitination and proteasomal-dependent degradation and CLC-2 is stabilized following lenalidomide treatment [84,85]. Crbn was identified as an epigenetic regulator of Kv1.3 K+ channels by promoting histone methylation at the *Kcna3* locus in murine CD4+ T-cells. Here, thalidomide treatment increased Kv1.3 expression [86].

Detailed structure–activity relationship studies of thalidomide in complex with CRL4^CRBN^ identified MEIS2 as an endogenous substrate that is stabilized in the presence of IMiD [6]. MEIS2 is a homeobox transcription factor that plays a key role in the maturation of cranial and cardiac neural crests [87]. MEIS2 is also associated with pro-survival activity in neuroblastoma and leukemia cells [88,89]. More recently, *JUN*, a well-studied proto-oncogene, was shown to be regulated by CRBN [90]. This observation is relevant in the context of LPS-induced inflammatory responses where CRBN dampens excessive proinflammatory cytokine expression induced by JUN/AP1 transcriptional activity [90]. However, further studies demonstrated that JUN levels are not altered upon IMiD exposure, as JUN binds to a CRBN domain that is distinct from that involved in substrate degradation [91]. CRBN also regulates NF-κB signaling in the context of an inflammatory response. Here CRBN binds to TRAF6, preventing its ubiquitination at K124, with subsequent reduction in TAB2 ubiquitination, NF-κB downregulation and dampened TLR4-dependent inflammation [92].

The alpha subunit of AMP kinase (AMPK), which contains the ATP-dependent catalytic domain, is an endogenous CRBN substrate that is stabilized by thalidomide [93]. AMPKα is a pivotal modulator of glucose and lipid metabolism; low AMPK activity enhances the Warburg effect and activates mTOR pathway signaling [94,95]. Other authors also report CRBN-dependent degradation of AMPKγ, another AMPK subunit [96]. The glucose transporter, GLUT1, is a CRBN-HSP90 client and its maturation is affected by IMiD treatment [9]. Together, these observations indicate that CRBN may regulate glucose metabolism. The resensitization of lenalidomide-resistance cells to IMiDs by deletion of the GID E3 ligase further supports this hypothesis [75]. Remarkably, CRBN also promotes degradation of glutamine synthase (GS) in a non-canonical manner. In the presence of high intracellular levels of glutamine, p300/CBP mediates acetylation of GS at lysine 11 and 14 [97]. Acetylated GS has an increased affinity for the thalidomide binding domain of CRBN. The recruitment of GS to CRBN is enhanced by IMiDs and CRBN-engagement mediates polyubiquitination and degradation of GS. Finally, resistance to IMiDs has been associated with the capacity of MM cells to resist the oxidative stress induced by IMiD treatment via inhibition of thioredoxin reductase (TXNR). An interaction between CRBN and thioredoxin (TXN) has been demonstrated, suggesting that CRBN may regulate the response to oxidative stress via disruption of the TXN-TXNR axis (Figure 1C) [98]. CRBN-dependent regulation of cellular metabolism is an interesting yet largely unexplored area. Given the multiplicity of metabolic pathways regulated by CRBN, we suggest that CISMs may have important consequences on cancer cell metabolism. 

## 9. Distinct and Overlapping Toxicity Profiles of the CISMs

Common side effects of therapy with IMiDs include peripheral neuropathy, venous thromboembolism and myelosuppression. Neuropathy is a greater issue in thalidomide-treated patients, usually arising within 12 months of drug commencement [99]. Thalidomide-induced peripheral neuropathy (TiPN) presents as chronic axonal neuropathy displaying Wallerian-like degeneration, without demyelination and in the absence of immune cell infiltrate [100]. Wallerian degeneration is cytokine driven, suggesting that thalidomide interferes with the inflammatory response to axonal damage [101,102]. Dysregulated cation channel function is also implicated in Wallerian degeneration [103,104], and thalidomide’s effects on Ca^++^ and K^+^ may therefore contribute to neuropathy [83,85,86]. However, the pathogenesis of TiPN remains poorly understood, and multiple events could perpetuate neurotoxicity. In contrast to neuropathy, myelosuppression is an uncommon thalidomide side effect [105] that is frequently observed with the more potent IMiDs. Myelosuppression has been attributed to IKZF1 degradation and subsequent downregulation of the transcription factor PU.1 [106,107,108]. As thalidomide is a much less potent IKZF1 degrader relative to lenalidomide and pomalidomide, it may not be as prone to this ‘on target’ toxicity [63]. Thrombocytopenia is a potential consequence of IMiD-induced aromatase degradation [109], which might also contribute to the reduction in platelet count in patients with del(5q) when treated with lenalidomide [52]. Thus, the distinct and overlapping side effect profile of the different CISMs (and their respective metabolites) may be explained by differential ‘on target’ effects on the CRL4^CRBN^ substrate/neosubstrate pool as well as modulation of other physiological CRBN functions (e.g., chaperone activity).

## 10. Rationally Developed CISMs: CELMoDs and Hetero-Bifunctional Targeted Protein Degraders

**Figure 3:**
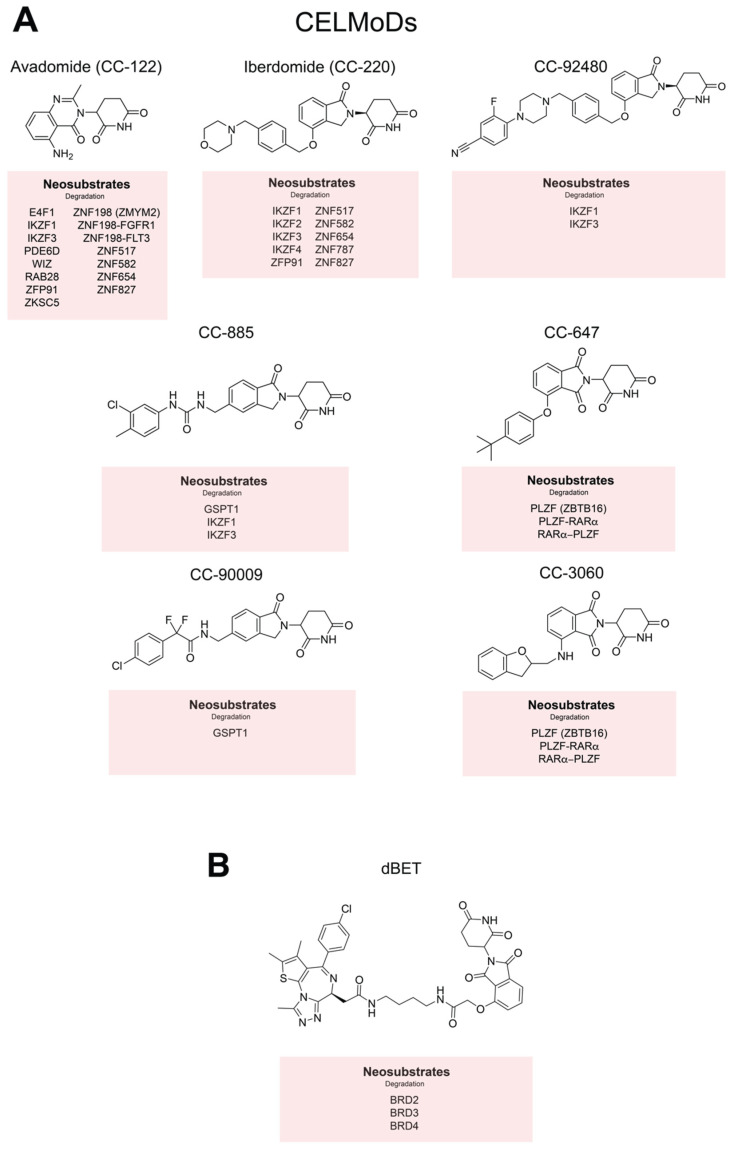
Cereblon E3 ligase modulators (CELMoDs), dBET and their respective protein targets. (**A**) The structure of avadomide, iberdomide, CC-92480, CC-885, CC-90009, CC-647 and CC-3060. Currently disclosed neosubstrates are listed in the red panels beneath. (**B**) The structure of the hetero-bifunctional selective degrader dBET and its targets.

CELMoDs, or Cereblon E3 ligase modulators, such as CC-885, CC-90009, avadomide, iberdomide, CC-92480, CC-3060 and CC-647 represent a new generation of ‘IMiDs’ which have been developed in the ‘post CRBN era’ (Figure 3A). CC-885 and CC-9009 target GSPT1, a translation termination factor. Degradation of GSPT1 is detrimental in acute myeloid leukemia (AML) cell lines and patient-derived AML samples [68]. CC-885 also induces IKZF1/3 degradation, whereas CC-90009 is highly specific for GSPT1 and leaves IKZF1 intact [110]. Analogous to CK1α, GSPT1 is engaged by CRBN despite the absence of ZF domains, again indicating the importance of protein tertiary structure in substrate binding. Avadomide does not degrade GSPT1, but is active on other canonical CRBN neosubstrates such as IKZF1, IKZF3 and ZFP91 [13,67]. Unlike other IMiDs or CELMoDs, avadomide degrades ZNF198 (or ZMYM2) fusion oncoproteins, such as ZNYM2-FGFR1 and ZMYM2-FLT3, indicating potential benefit for patients with acute lymphoblastic leukemias harboring these translocations [13]. CELMoDs CC-3060 and CC-647 degrade PLZF and the PLZF fusion proteins that are pathogenic in variant forms of promyelocytic leukemia. Both compounds target either PLZF-RARα or RARα-PLZF by engaging each at a distinct degron. Therefore, these molecules could be purposed therapeutically in the presence these uncommon cytogenetic entities [111]. Iberdomide is a CELMoD with demonstrable activity in MM patients who are refractory to pomalidomide [11,12]. Recent work demonstrated the activity of iberdomide across a panel of pomalidomide-resistant cell lines despite reduced CRBN expression [12]. The potency and acute kinetics of IKZF3 degradation are greater with iberdomide relative to pomalidomide, which may explain its activity in IMiD-refractory patients [49]. However, differential substrate specificity might also be at play, as iberdomide degrades IKZF2 and IKZF4 as well as IKZF1/3 [67]. Among the newest CELMoDs, CC-92480 also showed remarkable in vitro potency, degrading IKZF1/3 at concentrations as low as 1nM, and is now being assessed in IMiD-refractory myeloma [112].

Hetero-bifunctional targeted protein degraders hijack the function of E3 ubiquitin ligases to degrade specific vulnerabilities via the ubiquitin–proteasome system. This approach is particularly attractive where the use of simple enzymatic small molecules inhibitors is problematic [15,16]. Chimeric molecules interact with the binding receptor for an E3 ligase and a second desired protein target simultaneously and are referred to as ‘proteolysis-targeting chimeras’, or PROTACs [15]. CRBN is commonly utilized as a receptor for thalidomide-based degraders, where a phthalimide moiety is linked to a small molecule inhibitor against the desired target. In 2015, Winter et al. designed dBET1, a BET-bromodomain (BRD)-2/3/4 degrader (Figure 3B), by conjugating phthalimide to JQ1, an acetyl-lysine mimetic BRD2/4 inhibitor. The resulting molecule demonstrated superiority to JQ1 in reducing the tumor burden in murine models of acute leukemia [17]. Other attempts have been made to generate CRBN-based degraders for cancer vulnerabilities, including BCR-ABL selective degraders for Philadelphia-positive malignancies [113]. PROTAC chemistry is a rapidly evolving area of drug development and beyond the scope of this review to describe in detail. We direct the reader to recent reviews and the PROTAC-DB online database as contemporary resources [114,115,116].

## 11. Conclusions

**Figure 4:**
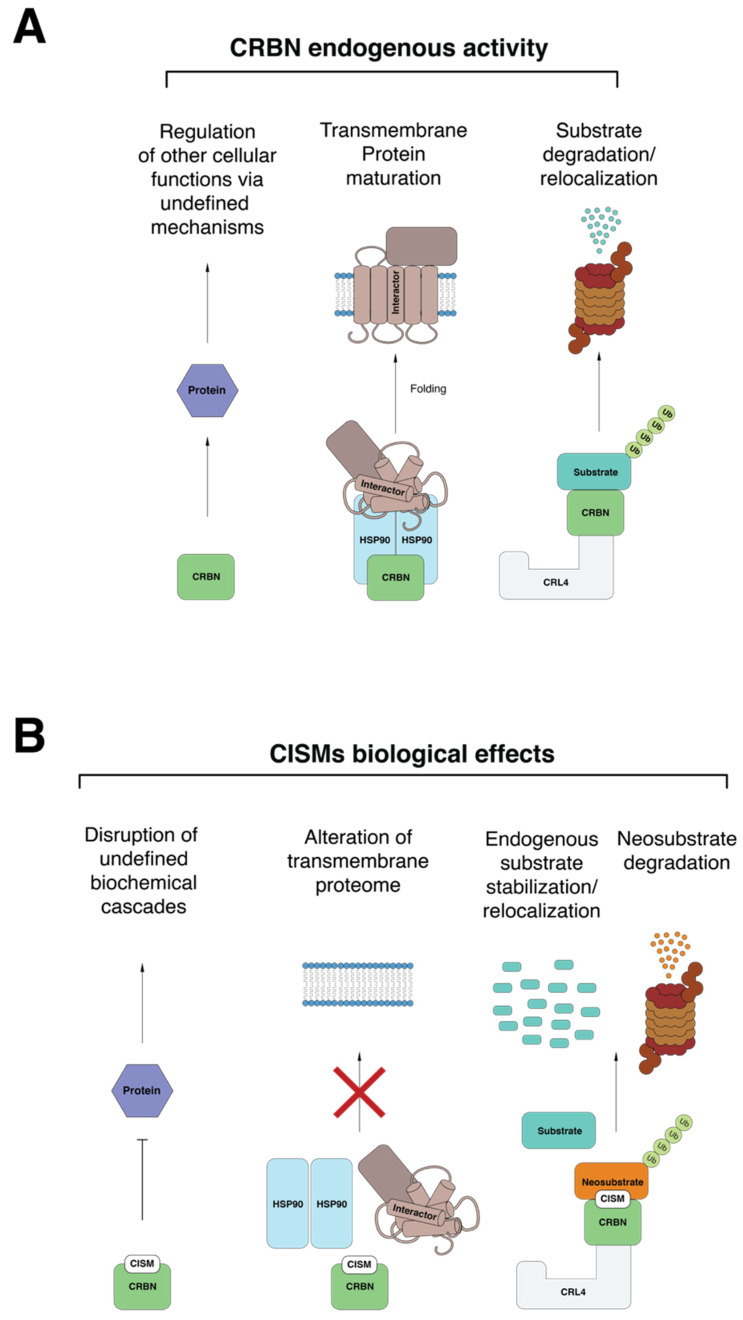
Intracellular pathways regulated by CRBN and potentially disrupted by treatment with CISMs. (**A**) CRBN interacts with the chaperone protein HSP90, augmenting the maturation of transmembrane proteins. CRBN is the adaptor of the E3 ligase CRL4 which engages proteins that are destined to the proteasome (e.g., MEIS2) or to other cell compartments (e.g., storage of BK Ca^++^ channels in the endoplasmic reticulum). CRBN also regulates other pathways via undefined mechanisms (e.g., responses to oxidative stress). (**B**) In the presence of a CISM, CRBNs endogenous targets may be inhibited (e.g., inhibition to the response to reactive oxygen species, transmembrane protein maturation), hijacked to ubiquitinate neosubstrates for proteasomal degradation (e.g., IKZF1, IKZF3) or accumulate in non-physiological cell compartments (e.g., redirection of BK Ca^++^ channels to surface membrane).

Following the initial identification of CRBN as the thalidomide binding protein by the Handa laboratory, the works of Krönke and Lu heralded the discovery that IMiDs modulate the ubiquitin ligase activity of CRL4^CRBN^. Together, the CISMs (thalidomide, IMiDs, CELMoDs and PROTACs) redirect CRL4^CRBN^ substrate specificity towards proteins that are normally not targeted by CRL4^CRBN^ [7,8] while stabilizing the expression of endogenous CRBN substrates [6]. After the first descriptions of canonical endogenous substrates (e.g., MEIS2) and neosubstrates (e.g., IKZF1/3, CK1α) a plethora of new CRL4^CRBN^ interactors have been discovered [63,64,67,84,85]. Differential structural configuration of various CISM chemotypes is proposed to be the basis of their different substrate specificity [66]. Differential potency between CISMs, the presence of active metabolites and variation in the specificity of engaged neosubstrates, likely underpins different side effects and tissue-specific toxicities. Given the number of biological processes regulated by CRBN (Figure 4A), the potential consequences of CISM treatment may be equally numerous. CISMs concurrently inhibit CRBN’s endogenous activity, engage neosubstrates for degradation, alter the transmembrane proteome and modulate important metabolic pathways in a fashion that is still not completely understood (Figure 4B). The anti-cancer and side effect profiles of CISMs are likely explained by the net product of these many biological events. CISMs exert their anti-myeloma action via distinct and complementary pathways, such as the induction of neosubstrate degradation, the inhibition of CRBN chaperone activity, and the modulation of the response to oxidative stress (summarized in Figure 1). The design and development of selective protein degraders may represent the quintessence of personalized medicine, as targeted protein degraders are theoretically capable of inducing degradation of any cancer vulnerability. The associated medicinal chemistry efforts now sit at a new threshold with CELMoDs and PROTACs being deployed in clinical trials [11,117]. The ability to rationally target a growing repertoire of cancer dependencies at the protein level will likely enable extension of the therapeutic landscape of CISMs well beyond their current niche in the myeloma clinic into the broader practice of precision oncology.

## References

[1] Kumar S.K., Rajkumar S.V., Dispenzieri A., Lacy M.Q., Hayman S.R., Buadi F.K., Zeldenrust S.R., Dingli D., Russell S.J., Lust J.A. (2008). Improved survival in multiple myeloma and the impact of novel therapies. Blood.

[2] Palumbo A., Anderson K. (2011). Multiple Myeloma. N. Engl. J. Med..

[3] Higgins J.J., Hao J., Kosofsky B.E., Rajadhyaksha A.M. (2008). Dysregulation of large-conductance Ca^2+^-activated K^+^ channel expression in nonsyndromal mental retardation due to a cereblon p.R419X mutation. Neurogenetics.

[4] Higgins J.J., Pucilowska J., Lombardi R.Q., Rooney J.P. (2004). A mutation in a novel ATP-dependent Lon protease gene in a kindred with mild mental retardation. Neurology.

[5] Ito T., Ando H., Suzuki T., Ogura T., Hotta K., Imamura Y., Yamaguchi Y., Handa H. (2010). Identification of a Primary Target of Thalidomide Teratogenicity. Science.

[6] Fischer E.S., Böhm K., Lydeard J.R., Yang H., Stadler M.B., Cavadini S., Nagel J., Serluca F., Acker V., Lingaraju G.M. (2014). Structure of the DDB1–CRBN E3 ubiquitin ligase in complex with thalidomide. Nature.

[7] Krönke J., Udeshi N.D., Narla A., Grauman P., Hurst S.N., McConkey M., Svinkina T., Heckl D., Comer E., Li X. (2014). Lenalidomide Causes Selective Degradation of IKZF1 and IKZF3 in Multiple Myeloma Cells. Science.

[8] Lu G., Middleton R.E., Sun H., Naniong M., Ott C.J., Mitsiades C.S., Wong K.-K., Bradner J.E., Kaelin W.G. (2014). The Myeloma Drug Lenalidomide Promotes the Cereblon-Dependent Destruction of Ikaros Proteins. Science.

[9] Heider M., Eichner R., Stroh J., Morath V., Kuisl A., Zecha J., Lawatscheck J., Baek K., Garz A.K., Rudelius M. (2021). The IMiD target CRBN determines HSP90 activity toward transmembrane proteins essential in multiple myeloma. Mol. Cell.

[10] Eichner R., Heider M., Fernández-Sáiz V., Van Bebber F., Garz A.K., Lemeer S., Rudelius M., Targosz B.S., Jacobs L., Knorn A.M. (2016). Immunomodulatory drugs disrupt the cereblon-CD147-MCT1 axis to exert antitumor activity and teratogenicity. Nat. Med..

[11] Lonial S., van de Donk N.W.C.J., Popat R., Zonder J.A., Minnema M.C., Larsen J., Nguyen T.V., Chen M.S., Bensmaine A., Cota M. (2019). First clinical (phase 1b/2a) study of iberdomide (CC-220; IBER), a CELMoD, in combination with dexamethasone (DEX) in patients (pts) with relapsed/refractory multiple myeloma (RRMM). J. Clin. Oncol..

[12] Bjorklund C.C., Kang J., Amatangelo M., Polonskaia A., Katz M., Chiu H., Couto S., Wang M., Ren Y., Ortiz M. (2019). Iberdomide (CC-220) is a potent cereblon E3 ligase modulator with antitumor and immunostimulatory activities in lenalidomide- and pomalidomide-resistant multiple myeloma cells with dysregulated CRBN. Leukemia.

[13] Renneville A., Gasser J.A., Grinshpun D.E., Beltran P.M.J., Tepper A., Guirguis A.A., Sellar R.S., Bristol-myers C. (2021). Avadomide induces degradation of ZMYM2 fusion oncoproteins in hematologic malignancies. Blood Cancer Discov..

[14] Huang X.V.D. (2016). Drugging the undruggables: Exploring the ubiquitin system for drug development. Cell Res..

[15] Lai A.C.C. (2017). Induced protein degradation: An emerging drug discovery paradigm. Nat. Rev. Drug Discov..

[16] Samarasinghe K.T.G., Crews C.M. (2021). Targeted protein degradation: A promise for undruggable proteins. Cell Chem. Biol..

[17] Winter G.E., Buckley D.L., Paulk J., Roberts J.M., Souza A., Dhe-Paganon S., Bradner J.E. (2015). Phthalimide conjugation as a strategy for in vivo target protein degradation. Science.

[18] Sampaio E.P., Sarno E.N., Galilly R., Cohn Z.A., Kaplan G. (1991). Thalidomide selectively inhibits tumor necrosis factor α production by stimulated human monocytes. J. Exp. Med..

[19] Corral L.G., Haslett P.A.J., Muller G.W., Chen R., Wong L.M., Ocampo C.J., Patterson R.T., Stirling D.I., Kaplan G. (1999). Differential cytokine modulation and T cell activation by two distinct classes of thalidomide analogues that are potent inhibitors of TNF-alpha. Int. J. Lepr. Other Mycobact. Dis..

[20] Payvandi F., Wu L., Haley M., Schafer P.H., Zhang L.H., Chen R.S., Muller G.W., Stirling D.I. (2004). Immunomodulatory drugs inhibit expression of cyclooxygenase-2 from TNF-α, IL-1β, and LPS-stimulated human PBMC in a partially IL-10-dependent manner. Cell. Immunol..

[21] Muller G.W., Chen R., Huang S.Y., Corral L.G., Wong L.M., Patterson R.T., Chen Y., Kaplan G., Stirling D.I. (1999). Amino-substituted thalidomide analogs: Potent inhibitors of TNF-α production. Bioorganic Med. Chem. Lett..

[22] Henry J.Y., Labarthe M.-C., Meyer B., Dasgupta P., Dalgleish A.G., Galustian C. (2013). Enhanced cross-priming of naive CD8+ T cells by dendritic cells treated by the IMiDs ® immunomodulatory compounds lenalidomide and pomalidomide. Immunology.

[23] Neuber B., Herth I., Tolliver C., Schoenland S., Hegenbart U., Hose D., Witzens-Harig M., Ho A.D., Goldschmidt H., Klein B. (2011). Lenalidomide Enhances Antigen-Specific Activity and Decreases CD45RA Expression of T Cells from Patients with Multiple Myeloma. J. Immunol..

[24] Neuber B., Dai J., Waraich W.A., Awwad M.H.S., Engelhardt M., Schmitt M., Medenhoff S., Harig M.W., Ho A.D., Goldschmidt H. (2017). Lenalidomide overcomes the immunosuppression of regulatory CD8+CD28- T-cells. Oncotarget.

[25] Quintana F.J., Jin H., Burns E.J., Nadeau M., Yeste A., Kumar D., Rangachari M., Zhu C., Xiao S., Seavitt J. (2012). Aiolos promotes T H17 differentiation by directly silencing Il2 expression. Nat. Immunol..

[26] Galustian C., Meyer B., Labarthe M.-C., Dredge K., Klaschka D., Henry J., Todryk S., Chen R., Muller G., Stirling D. (2008). The anti-cancer agents lenalidomide and pomalidomide inhibit the proliferation and function of T regulatory cells. Cancer Immunol. Immunother..

[27] Lagrue K., Carisey A., Morgan D.J., Chopra R., Davis D.M. (2015). Lenalidomide augments actin remodeling and lowers NK-cell activation thresholds. Blood.

[28] Hsu A.K., Quach H., Tai T., Prince H.M., Harrison S.J., Trapani J.A., Smyth M.J., Neeson P., Ritchie D.S. (2011). The immunostimulatory effect of lenalidomide on NK-cell function is profoundly inhibited by concurrent dexamethasone therapy. Blood.

[29] Wu L., Parton A., Lu L., Adams M., Schafer P., Bartlett J.B. (2011). Lenalidomide enhances antibody-dependent cellular cytotoxicity of solid tumor cells in vitro: Influence of host immune and tumor markers. Cancer Immunol. Immunother..

[30] D’Amato R.J., Loughnan M.S., Flynn E., Folkman J. (1994). Thalidomide is an inhibitor of angiogenesis. Proc. Natl. Acad. Sci. USA.

[31] Yabu T., Tomimoto H., Taguchi Y., Yamaoka S., Igarashi Y., Okazaki T. (2005). Thalidomide-induced antiangiogenic action is mediated by ceramide through depletion of VEGF receptors, and is antagonized by sphingosine-1-phosphate. Blood.

[32] De Luisi A., Ferrucci A., Coluccia A.M.L., Ria R., Moschetta M., de Luca E., Pieroni L., Maffia M., Urbani A., Di Pietro G. (2011). Lenalidomide Restrains Motility and Overangiogenic Potential of Bone Marrow Endothelial Cells in Patients with Active Multiple Myeloma. Clin. Cancer Res..

[33] Lu L., Payvandi F., Wu L., Zhang L.-H., Hariri R.J., Man H.-W., Chen R.S., Muller G.W., Hughes C.C.W., Stirling D.I. (2009). The anti-cancer drug lenalidomide inhibits angiogenesis and metastasis via multiple inhibitory effects on endothelial cell function in normoxic and hypoxic conditions. Microvasc. Res..

[34] Anderson K.C. (2005). Lenalidomide and thalidomide: Mechanisms of action—similarities and differences. Semin. Hematol..

[35] Bolzoni M., Storti P., Bonomini S., Todoerti K., Guasco D., Toscani D., Agnelli L., Neri A., Rizzoli V., Giuliani N. (2013). Immunomodulatory drugs lenalidomide and pomalidomide inhibit multiple myeloma-induced osteoclast formation and the RANKL/OPG ratio in the myeloma microenvironment targeting the expression of adhesion molecules. Exp. Hematol..

[36] Geitz H., Handt S., Zwingenberger K. (1996). Thalidomide selectively modulates the density of cell surface molecules involved in the adhesion cascade. Immunopharmacology.

[37] Gandhi A.K., Kang J., Capone L., Parton A., Wu L., Zhang L.H., Mendy D., Lopez-Girona A., Tran T., Sapinoso L. (2010). Dexamethasone Synergizes with Lenalidomide to Inhibit Multiple Myeloma Tumor Growth, But Reduces Lenalidomide-Induced Immunomodulation of T and NK Cell Function. Curr. Cancer Drug Targets.

[38] Verhelle D., Corral L.G., Wong K., Mueller J.H., Moutouh-De Parseval L., Jensen-Pergakes K., Schafer P.H., Chen R., Glezer E., Ferguson G.D. (2007). Lenalidomide and CC-4047 Inhibit the Proliferation of Malignant B Cells while Expanding Normal CD34+ Progenitor Cells. Cancer Res..

[39] Lopez-Girona A., Heintel D., Zhang L.-H., Mendy D., Gaidarova S., Brady H., Bartlett J.B., Schafer P.H., Schreder M., Bolomsky A. (2011). Lenalidomide downregulates the cell survival factor, interferon regulatory factor-4, providing a potential mechanistic link for predicting response. Br. J. Haematol..

[40] Keifer J.A., Guttridge D.C., Ashburner B.P., Baldwin A.S. (2001). Inhibition of NF-κB Activity by Thalidomide through Suppression of IκB Kinase Activity. J. Biol. Chem..

[41] Mitsiades N., Mitsiades C.S., Poulaki V., Chauhan D., Richardson P.G., Hideshima T., Munshi N., Treon S.P., Anderson K.C. (2002). Biologic sequelae of nuclear factor-κB blockade in multiple myeloma: Therapeutic applications. Blood.

[42] Zhu Y.X., Braggio E., Shi C.-X., Bruins L.A., Schmidt J.E., Van Wier S., Chang X.-B., Bjorklund C.C., Fonseca R., Bergsagel P.L. (2011). Cereblon expression is required for the antimyeloma activity of lenalidomide and pomalidomide. Blood.

[43] Heintel D., Rocci A., Ludwig H., Bolomsky A., Caltagirone S., Schreder M., Pfeifer S., Gisslinger H., Zojer N., Jäger U. (2013). High expression of cereblon (CRBN) is associated with improved clinical response in patients with multiple myeloma treated with lenalidomide and dexamethasone. Br. J. Haematol..

[44] Maity R., Neri P.E., Tagoug I., Ren L., Slaby J., Jimenez-Zepeda V.H., Duggan P., Simms J., Bahlis N.J. (2014). Cereblon (CRBN) Splice Isoform Lacking Exon 10 Attenuates Lenalidomide-Mediated Degradation of Aiolos and Is Upregulated in Immunomodulatory Drugs (IMiDs) Resistant Myeloma (MM) Patients. Blood.

[45] Gooding S., Ansari-Pour N., Towfic F., Estévez M.O., Chamberlain P.P., Tsai K.T., Flynt E., Hirst M., Rozelle D., Dhiman P. (2021). Multiple cereblon genetic changes are associated with acquired resistance to lenalidomide or pomalidomide in multiple myeloma. Blood.

[46] Shaffer A.L., Emre N.C.T., Lamy L., Ngo V.N., Wright G., Xiao W., Powell J., Dave S., Yu X., Zhao H. (2008). IRF4 addiction in multiple myeloma. Nature.

[47] Holien T., Våtsveen T.K., Hella H., Waage A., Sundan A. (2012). Addiction to c-MYC in multiple myeloma. Blood.

[48] Raisner R., Kharbanda S., Jin L., Jeng E., Chan E., Merchant M., Haverty P.M., Bainer R., Cheung T., Arnott D. (2018). Enhancer Activity Requires CBP/P300 Bromodomain-Dependent Histone H3K27 Acetylation. Cell Rep..

[49] Bjorklund C.C., Lu L., Kang J., Hagner P.R., Havens C.G., Amatangelo M., Wang M., Ren Y., Couto S., Breider M. (2015). Rate of CRL4CRBN substrate Ikaros and Aiolos degradation underlies differential activity of lenalidomide and pomalidomide in multiple myeloma cells by regulation of c-Myc and IRF4. Blood Cancer J..

[50] Patil A., Manzano M., Gottwein E. (2018). CK1a and IRF4 are essential and independent effectors of immunomodulatory drugs in primary effusion lymphoma. Blood.

[51] Gandhi A.K., Kang J., Havens C.G., Conklin T., Ning Y., Wu L., Ito T., Ando H., Waldman M.F., Thakurta A. (2014). Immunomodulatory agents lenalidomide and pomalidomide co-stimulate T cells by inducing degradation of T cell repressors Ikaros and Aiolos via modulation of the E3 ubiquitin ligase complex CRL4CRBN. Br. J. Haematol..

[52] List A., Dewald G., Bennett J., Giagounidis A., Raza A., Feldman E., Powell B., Greenberg P., Thomas D., Stone R. (2006). Lenalidomide in the Myelodysplastic Syndrome with Chromosome 5q Deletion. N. Engl. J. Med..

[53] Ebert B.L., Pretz J., Bosco J., Chang C.Y., Tamayo P., Galili N., Raza A., Root D.E., Attar E., Ellis S.R. (2008). Identification of RPS14 as a 5q- syndrome gene by RNA interference screen. Nature.

[54] Dutt S., Narla A., Lin K., Mullally A., Abayasekara N., Megerdichian C., Wilson F.H., Currie T., Khanna-Gupta A., Berliner N. (2011). Haploinsufficiency for ribosomal protein genes causes selective activation of p53 in human erythroid progenitor cells. Blood.

[55] Barlow J.L., Drynan L.F., Hewett D.R., Holmes L.R., Lorenzo-Abalde S., Lane A.L., Jolin H.E., Pannell R., Middleton A.J., Wong S.H. (2009). A p53-dependent mechanism underlies macrocytic anemia in a mouse model of human 5q-syndrome. Nat. Med..

[56] Kumar M.S., Narla A., Nonami A., Mullally A., Dimitrova N., Ball B., McAuley J.R., Poveromo L., Kutok J.L., Galili N. (2011). Coordinate loss of a microRNA and protein-coding gene cooperate in the pathogenesis of 5q syndrome. Blood.

[57] Shortt J., Hsu A.K., Johnstone R.W. (2013). Thalidomide-analogue biology: Immunological, molecular and epigenetic targets in cancer therapy. Oncogene.

[58] Krönke J., Fink E.C., Hollenbach P.W., MacBeth K.J., Hurst S.N., Udeshi N.D., Chamberlain P.P., Mani D.R., Man H.W., Gandhi A.K. (2015). Lenalidomide induces ubiquitination and degradation of CK1α in del(5q) MDS. Nature.

[59] Hu Y., Song W., Cirstea D., Lu D., Munshi N.C., Anderson K.C. (2015). CSNK1α1 mediates malignant plasma cell survival. Leukemia.

[60] Manni S., Carrino M., Manzoni M., Gianesin K., Nunes S.C., Costacurta M., Tubi L.Q., Macaccaro P., Taiana E., Cabrelle A. (2017). Inactivation of CK1α in multiple myeloma empowers drug cytotoxicity by affecting AKT and ß-catenin survival signaling pathways. Oncotarget.

[61] Carrino M., Quotti Tubi L., Fregnani A., Canovas Nunes S., Barilà G., Trentin L., Zambello R., Semenzato G., Manni S., Piazza F. (2019). Prosurvival autophagy is regulated by protein kinase CK1 alpha in multiple myeloma. Cell Death Discov..

[62] An J., Ponthier C.M., Sack R., Seebacher J., Stadler M.B., Donovan K.A., Fischer E.S. (2017). PSILAC mass spectrometry reveals ZFP91 as IMiD-dependent substrate of the CRL4 CRBN ubiquitin ligase. Nat. Commun..

[63] Donovan K.A., An J., Nowak R.P., Yuan J.C., Fink E.C., Berry B.C., Ebert B.L., Fischer E.S. (2018). Thalidomide promotes degradation of SALL4, a transcription factor implicated in Duane radial ray syndrome. eLife.

[64] Yamanaka S., Murai H., Saito D., Abe G., Tokunaga E., Iwasaki T., Takahashi H., Takeda H., Suzuki T., Shibata N. (2021). Thalidomide and its metabolite 5-hydroxythalidomide induce teratogenicity via the cereblon neosubstrate PLZF. EMBO J..

[65] Asatsuma-Okumura T., Ando H., De Simone M., Yamamoto J., Sato T., Shimizu N., Asakawa K., Yamaguchi Y., Ito T., Guerrini L. (2019). P63 Is a Cereblon Substrate Involved in Thalidomide Teratogenicity. Nat. Chem. Biol..

[66] Chamberlain P.P., Lopez-Girona A., Miller K., Carmel G., Pagarigan B., Chie-Leon B., Rychak E., Corral L.G., Ren Y.J., Wang M. (2014). Structure of the human Cereblon-DDB1-lenalidomide complex reveals basis for responsiveness to thalidomide analogs. Nat. Struct. Mol. Biol..

[67] Sievers Q.L., Petzold G., Bunker R.D., Renneville A., Słabicki M., Liddicoat B.J., Abdulrahman W., Mikkelsen T., Ebert B.L., Thomä N.H. (2018). Defining the human C_2_H_2_ zinc finger degrome targeted by thalidomide analogs through CRBN. Science.

[68] Matyskiela M.E., Lu G., Ito T., Pagarigan B., Lu C.C., Miller K., Fang W., Wang N.Y., Nguyen D., Houston J. (2016). A novel cereblon modulator recruits GSPT1 to the CRL4 CRBN ubiquitin ligase. Nature.

[69] Mali P., Yang L., Esvelt K.M., Aach J., Guell M., DiCarlo J.E., Norville J.E., Church G.M. (2013). RNA-guided human genome engineering via Cas9. Science.

[70] Joung J., Konermann S., Gootenberg J.S., Abudayyeh O.O., Platt R.J., Brigham M.D., Sanjana N.E., Zhang F. (2017). Genome-scale CRISPR-Cas9 knockout and transcriptional activation screening. Nat. Protoc..

[71] Shalem O., Sanjana N.E., Hartenian E., Shi X., Scott D.A., Mikkelsen T.S., Heckl D., Ebert B.L., Root D.E., Doench J.G. (2014). Genome-scale CRISPR-Cas9 knockout screening in human cells. Science.

[72] Cluse L.A., Nikolic I., Knight D., Madhamshettiwar P.B., Luu J., Cowley K.J., Semple T., Mir Arnau G., Shortt J., Johnstone R.W. (2018). A Comprehensive Protocol Resource for Performing Pooled shRNA and CRISPR Screens. Methods Mol. Biol..

[73] Sievers Q.L., Gasser J.A., Cowley G.S., Fischer E.S., Ebert B.L. (2018). Genome-wide screen identifies cullin-RING ligase machinery required for lenalidomide-dependent CRL4CRBN activity. Blood.

[74] Liu J., Song T., Zhou W., Xing L., Wang S., Ho M., Peng Z., Tai Y.-T., Hideshima T., Anderson K.C. (2019). A genome-scale CRISPR-Cas9 screening in myeloma cells identifies regulators of immunomodulatory drug sensitivity. Leukemia.

[75] Costacurta M., Vervoort S.J., Hogg S.J., Martin B.P., Johnstone R.W., Shortt J. (2021). Whole genome CRISPR screening identifies TOP2B as a potential target for IMiD sensitization in multiple myeloma. Haematologica.

[76] Lu G., Weng S., Matyskiela M., Zheng X., Fang W., Wood S., Surka C., Mizukoshi R., Lu C.C., Mendy D. (2018). UBE2G1 governs the destruction of cereblon neomorphic substrates. eLife.

[77] Zhou W., Xu J., Tan M., Li H., Li H., Wei W., Sun Y. (2018). UBE2M Is a Stress-Inducible Dual E2 for Neddylation and Ubiquitylation that Promotes Targeted Degradation of UBE2F. Mol. Cell.

[78] Cavadini S., Fischer E.S., Bunker R.D., Potenza A., Lingaraju G.M., Goldie K.N., Mohamed W.I., Faty M., Petzold G., Beckwith R.E.J. (2016). Cullin-RING ubiquitin E3 ligase regulation by the COP9 signalosome. Nature.

[79] Lingaraju G.M., Bunker R.D., Cavadini S., Hess D., Hassiepen U., Renatus M., Fischer E.S., Thomä N.H. (2014). Crystal structure of the human COP9 signalosome. Nature.

[80] Zhou L., Xu G. (2019). Cereblon attenuates DNA damage-induced apoptosis by regulating the transcription-independent function of p53. Cell Death Dis..

[81] Zhu Y.X., Shi C.X., Bruins L.A., Wang X., Riggs D.L., Porter B., Ahmann J.M., de Campos C.B., Braggio E., Bergsagel P.L. (2019). Identification of lenalidomide resistance pathways in myeloma and targeted resensitization using cereblon replacement, inhibition of STAT3 or targeting of IRF4. Blood Cancer J..

[82] Hogg S.J., Motorna O., Cluse L.A., Johanson T.M., Coughlan H.D., Raviram R., Myers R.M., Costacurta M., Todorovski I., Pijpers L. (2021). Targeting histone acetylation dynamics and oncogenic transcription by catalytic P300/CBP inhibition. Mol. Cell.

[83] Song T., Liang S., Liu J., Zhang T., Yin Y., Geng C., Gao S., Feng Y., Xu H., Guo D. (2018). CRL4 antagonizes SCFFbxo7-mediated turnover of cereblon and BK channel to regulate learning and memory. PLoS Genet..

[84] Chen Y.-A., Peng Y.-J., Hu M.-C., Huang J.-J., Chien Y.-C., Wu J.-T., Chen T.-Y., Tang C.-Y. (2015). The Cullin 4A/B-DDB1-Cereblon E3 Ubiquitin Ligase Complex Mediates the Degradation of CLC-1 Chloride Channels. Sci. Rep..

[85] Fu S.J., Hu M.C., Peng Y.J., Fang H.Y., Hsiao C.T., Chen T.Y., Jeng C.J., Tang C.Y. (2020). CUL4-DDB1-CRBN E3 Ubiquitin Ligase Regulates Proteostasis of ClC-2 Chloride Channels: Implication for Aldosteronism and Leukodystrophy. Cells.

[86] Kang J.-A., Park S.-H., Jeong S.P., Han M.-H., Lee C.-R., Lee K.M., Kim N., Song M.-R., Choi M., Ye M. (2016). Epigenetic regulation of Kcna3-encoding Kv1.3 potassium channel by cereblon contributes to regulation of CD4+ T-cell activation. Proc. Natl. Acad. Sci. USA.

[87] Machon O., Masek J., Machonova O., Krauss S., Kozmik Z. (2015). Meis2 is essential for cranial and cardiac neural crest development. BMC Dev. Biol..

[88] Lai C.K., Norddahl G.L., Maetzig T., Rosten P., Lohr T., Sanchez Milde L., von Krosigk N., Docking T.R., Heuser M., Karsan A. (2017). Meis2 as a critical player in MN1-induced leukemia. Blood Cancer J..

[89] Zha Y., Xia Y., Ding J., Choi J.H., Yang L., Dong Z., Yan C., Huang S., Ding H.F. (2014). MEIS2 is essential for neuroblastoma cell survival and proliferation by transcriptional control of M-phase progression. Cell Death Dis..

[90] Yang J., Huang M., Zhou L., He X., Jiang X., Zhang Y., Xu G. (2018). Cereblon suppresses the lipopolysaccharide-induced inflammatory response by promoting the ubiquitination and degradation of c-Jun. J. Biol. Chem..

[91] Tao J., Yang J., Xu G. (2018). The interacting domains in cereblon differentially modulate the immunomodulatory drug-mediated ubiquitination and degradation of its binding partners. Biochem. Biophys. Res. Commun..

[92] Min Y., Wi S.M., Kang J.-A., Yang T., Park C.-S., Park S.-G., Chung S., Shim J.-H., Chun E., Lee K.-Y. (2016). Cereblon negatively regulates TLR4 signaling through the attenuation of ubiquitination of TRAF6. Cell Death Dis..

[93] Kwon E., Li X., Deng Y., Chang H.W., Kim D.Y. (2019). AMPK is down-regulated by the CRL4A-CRBN axis through the polyubiquitination of AMPKα isoforms. FASEB J..

[94] Faubert B., Boily G., Izreig S., Griss T., Samborska B., Dong Z., Dupuy F., Chambers C., Fuerth B.J., Viollet B. (2013). AMPK Is a Negative Regulator of the Warburg Effect and Suppresses Tumor Growth In Vivo. Cell Metab..

[95] Shackelford D.B., Shaw R.J. (2009). The LKB1–AMPK pathway: Metabolism and growth control in tumour suppression. Nat. Rev. Cancer.

[96] Yang S.J., Jeon S.J., Van Nguyen T., Deshaies R.J., Park C.S., Lee K.M. (2020). Ubiquitin-dependent proteasomal degradation of AMPK gamma subunit by Cereblon inhibits AMPK activity. Biochim. Biophys. Acta Mol. Cell Res..

[97] Van Nguyen T., Lee J.E., Sweredoski M.J., Yang S.J., Jeon S.J., Harrison J.S., Yim J.H., Lee S.G., Handa H., Kuhlman B. (2016). Glutamine Triggers Acetylation-Dependent Degradation of Glutamine Synthetase via the Thalidomide Receptor Cereblon. Mol. Cell.

[98] Arnér E.S.J., Holmgren A. (2000). Physiological functions of thioredoxin and thioredoxin reductase. Eur. J. Biochem..

[99] Mileshkin L., Stark R., Day B., Seymour J.F., Zeldis J.B., Prince H.M. (2006). Development of Neuropathy in Patients With Myeloma Treated With Thalidomide: Patterns of Occurrence and the Role of Electrophysiologic Monitoring. J. Clin. Oncol..

[100] Chaudhry V., Cornblath D.R., Corse A., Freimer M., Simmons-O’Brien E., Vogelsang G. (2002). Thalidomide-induced neuropathy. Neurology.

[101] Liefner M., Siebert H., Sachse T., Michel U., Kollias G., Brück W. (2000). The role of TNF-α during Wallerian degeneration. J. Neuroimmunol..

[102] Shamash S., Reichert F., Rotshenker S. (2002). The cytokine network of wallerian degeneration: Tumor necrosis factor-α, interleukin-1α, and interleukin-1β. J. Neurosci..

[103] Mishra B., Carson R., Hume R.I., Collins C.A. (2013). Sodium and potassium currents influence wallerian degeneration of injured Drosophila axons. J. Neurosci..

[104] Loreto A., Di Stefano M., Gering M., Conforti L. (2015). Wallerian Degeneration Is Executed by an NMN-SARM1-Dependent Late Ca2+ Influx but Only Modestly Influenced by Mitochondria. Cell Rep..

[105] Wang M., Dimopoulos M.A., Chen C., Cibeira M.T., Attal M., Spencer A., Rajkumar S.V., Yu Z., Olesnyckyj M., Zeldis J.B. (2008). Lenalidomide plus dexamethasone is more effective than dexamethasone alone in patients with relapsed or refractory multiple myeloma regardless of prior thalidomide exposure. Blood.

[106] Pal R., Monaghan S.A., Hassett A.C., Mapara M.Y., Schafer P., Roodman G.D., Ragni M.V., Moscinski L., List A., Lentzsch S. (2010). Immunomodulatory derivatives induce PU.1 down-regulation, myeloid maturation arrest, and neutropenia. Blood.

[107] Liu A., Li S., Donnenberg V., Fu J., Gollin S.M., Ma H., Lu C., Stolz D.B., Mapara M.Y., Monaghan S.A. (2018). Immunomodulatory drugs downregulate IKZF1 leading to expansion of hematopoietic progenitors with concomitant block of megakaryocytic maturation. Haematologica.

[108] Li S., Fu J., Wang H., Ma H., Xu X., Yang Y.-G., Deng S., Mapara M.Y., Lentzsch S. (2018). IMiD compounds affect CD34+ cell fate and maturation via CRBN-induced IKZF1 degradation. Blood Adv..

[109] Tochigi T., Miyamoto T., Hatakeyama K., Sakoda T., Ishihara D., Irifune H., Shima T., Kato K., Maeda T., Ito T. (2020). Aromatase is a novel neosubstrate of cereblon responsible for immunomodulatory drug–induced thrombocytopenia. Blood.

[110] Hansen J.D., Correa M., Alexander M., Nagy M., Huang D., Sapienza J., Lu G., Lebrun L.A., Cathers B.E., Zhang W. (2021). CC-90009: A Cereblon E3 Ligase Modulating Drug That Promotes Selective Degradation of GSPT1 for the Treatment of Acute Myeloid Leukemia. J. Med. Chem..

[111] Matyskiela M.E., Zhu J., Baughman J.M., Clayton T., Slade M., Wong H.K., Danga K., Zheng X., Labow M., Lebrun L. (2020). Cereblon Modulators Target ZBTB16 and Its Oncogenic Fusion Partners for Degradation via Distinct Structural Degrons. ACS Chem. Biol..

[112] Hansen J.D., Correa M., Nagy M.A., Alexander M., Plantevin V., Grant V., Whitefield B., Huang D., Kercher T., Harris R. (2020). Discovery of CRBN E3 Ligase Modulator CC-92480 for the Treatment of Relapsed and Refractory Multiple Myeloma. J. Med. Chem..

[113] Lai A.C., Toure M., Hellerschmied D., Salami J., Jaime-Figueroa S., Ko E., Hines J., Crews C.M. (2016). Modular PROTAC Design for the Degradation of Oncogenic BCR-ABL. Angew. Chemie Int. Ed..

[114] Hu Z., Crews C.M. (2021). Recent Developments in PROTAC-Mediated Protein Degradation: From Bench to Clinic. ChemBioChem.

[115] Bricelj A., Steinebach C., Kuchta R., Gütschow M., Sosič I. (2021). E3 Ligase Ligands in Successful PROTACs: An Overview of Syntheses and Linker Attachment Points. Front. Chem..

[116] Weng G., Shen C., Cao D., Gao J., Dong X., He Q., Yang B., Li D., Wu J., Hou T. (2021). PROTAC-DB: An online database of PROTACs. Nucleic Acids Res..

[117] Li X., Song Y. (2020). Proteolysis-targeting chimera (PROTAC) for targeted protein degradation and cancer therapy. J. Hematol. Oncol..

